# Determination of Pentraxin-3, Interleukin-8 and Vascular Endothelial Growth Factor Levels in Patients with Gastric Adenocarcinoma

**DOI:** 10.31557/APJCP.2021.22.5.1507

**Published:** 2021-05

**Authors:** Mustafa Yeni, Ercan Korkut, Nurhak Aksungur, Salih Kara, Seda Askin, Murat Kartal

**Affiliations:** 1 *General Surgery Clinic, Regional Training and Research Hospital, Erzurum, Turkey. *; 2 *Department of General Surgery, Atatürk University Faculty of Medicine, Erzurum, Turkey. *; 3 *General Surgery, Faculty of Medicine, Ataturk University, Erzurum, Turkey. *; 4 *Department of Biochemistry, Atatürk University, Erzurum, Turkey. *; 5 *Department of General Surgery, Erzurum Regional Training Research Hospital, Erzurum, Turkey. *

**Keywords:** Gastric adenocarcinoma, Pentraxin-3- IL-8, VEGF

## Abstract

**Introduction and Aim:**

The purpose of this study was to determine the value, in terms of diagnosis, resectability and prognosis of pentraxin-3 (PTX3), interleukin-8 (IL-8) and vascular endothelial growth factor (VEGF) in cases of gastric adenocarcinoma, an important condition both worldwide and in Turkey, and to determine their levels in order to contribute to elucidating the pathogenesis of the disease.

**Materials and Methods:**

Serum was separated from blood specimens collected from 45 patients diagnosed with gastric adenocarcinoma and from a 30-member healthy control group. Serum PTX3, IL-8 and VEGF levels were studied by ELISA method.

**Results:**

Serum PTX3 values differed significantly between the patient group and the control group (p<0.05). Serum IL-8 values also differed significantly between the patient group and the control group (p<0.05). A significant difference was also observed between serum VEGF values in the patient group and the control group (p<0.05). Significant correlation was determined between serum PTX3 and VEGF (p<0.01; r=0.833), between serum PTX3 and IL-8 (p<0.01; r=0.818), and between serum VEGF and IL-8 (p<0.01; r=0.803), measurements when the entire study population was evaluated irrespectively of groups.

**Conclusion:**

Serum PTX3, IL-8 and VEGF levels decreased in cases of gastric adenocarcinoma compared to the control group, and their levels affected one another.

## Introduction

Gastric cancer is a major health problem all over the world. Due to Cancer Statistics 2020 report, 27,600 new gastric cancer cases are thought to be seen. Also, it is estimated that 11,010 people will die due to gastric cancer in the USA (Siegel et al., 2020). Approximately 90-95% of tumors in the stomach are malignant. Ninety-five percent of these consist of carcinomas, while the remaining 5% are made up of 75% lymphomas and 25% malignant mesenchymal tumors. Diet-related environmental factors have been largely implicated in the etiology of gastric cancer. The best known of these include foods preserved by smoking, salting, or nitrates. Cigarettes, which contain numerous carcinogenic agents, and probably alcohol, are other risk factors. In contrast, fresh fruit and vegetables, a fiber-rich diet, vitamin C, and probably also vitamin E and beta-carotene, have been reported to exhibit protective effects. Genetic and congenital factors have also been implicated in gastric cancers. This risk is at least twice as high in the primary relatives of gastric cancer patients. Blood group A is another known risk factor (Cheng et al., 2016).

Pentraxin-3(PTX3) is a member of thepentraxin family and is the active form of pentraxin. PTX-3 is released from macrophages and other types of cell as a result of stimulation of primary inflammatory mediators such as lipopolysaccharides, interleukin-1 (IL-1) and tumor necrosis factor-α (TNF-α). In-vitro studies have shown that various types of cell release PTX3 as a primary inflammatory signal mediator. Plasma levels of PTX3 are quite low (2 ng/ml) but increase in certain pathological events involving inflammation (Aksungur et al., 2015).

Interleukin-8 (IL-8) is a member of a broad family consisting of low molecular weight cytokines known as chemokines. IL-8 is important in inflammation and cell migration and is particularly produced in endothelial cells and macrophages. It was originally thought to be a neutron-chemotactic factor but has also been identified as a chemotactic factor for basophils, T lymphocytes, and natural killer cells (Mukado et al., 1995).

Vascular endothelial growth factor (VEGF), a member of the platelet-derived growth factor superfamily, is specific to endothelial cells. It plays a role in both physiological events, and also in several pathological events, including tumor growth and spread. VEGFproduction is initiated by various factors, including platelet-derived growth factor-BB, TNF-α, keratinocyte growth factor transforming growth factor-β1, epidermal growth factor (EGF), and interleukin -β1. Hypoxia is perhaps one of the most important stimuli inducing the synthesis of VEGF and its receptors (Yazır et al., 2004).

## Materials and Methods

Approval for this study was granted by the Erzurum Atatürk University Medical Faculty ethical committee (no. 1500139240 dated 29.12.2015). The study involved patients hospitalized in the Atatürk University Medical Faculty General Surgery Clinic, Turkey, between January 2016 and January 2018 with diagnoses of gastric adenocarcinoma. Forty-five adult patients diagnosed with gastric adenocarcinoma based on pathology reports and 30 healthy individuals were enrolled. Patients aged over 18 were randomly selected. The subjects of the control group were normotensive and had no systemic disease. After an overnight fasting, 10 ml of blood was drawn by venipucture into biochemical glass tubes. Blood samples were centrifuged at 2,000 x g for 10 min and the serum samples obtained were stored at -80°C until assayed. Serum PTX3, VEGF and IL-8 levels were determined by the ELISA method using commercial kits (Kit name: Sunredbio Human Pentraxin-3(PTX3) ELISA Kit, Sunredbio Vascular Endothelial Cell Growth Factor (VEGF), Sunredbio Human Interleukin-8 (IL-8) ELISA Kit, Cat No: 201-12-1939, 201-12-0081, 201-12-0090, Country: China, respectively). 

*Statistical Analysis*

Data analysis was performed on SPSS 22.0 software. Descriptive statistics were expressed as number and percentage for categorical data and as mean plus standard deviation for numerical data. Normality of distribution of numerical variables was examined using a complete blood count chart. Student’s t test, the Mann-Whitney U test, and Spearman’s correlation test were employed as hypothesis tests. A p value of less than 0.05 was considered to be a statistically significant.

## Results

A 45-member patient group (60%) diagnosed with gastric adenocarcinoma and a 30-member healthy control group (40%) were enrolled in this study. The patient group consisted of 34 men (76%) and 11 women (24%), and the healthy control group of 20 men (66%) and 10 women (33%). No significant difference was determined between the patient and control groups in terms of sex (p>0.05). The mean age of the individuals in the study was 48.15±19.75 years (30-84), and the mean age of the patient group was 61.76±12. Twenty-three tumors were localized distally (51.1%) and 22 proximally (49%). In terms of blood groups, 27 patients (60%) were A Rh (+) and 11 (25%) were 0 Rh (+), and the great majority of patients belonged to these two groups. Forty-two patients (93.3%) were from the Rh (+) subgroup. Serum PTX3, VEGF and IL-8 measurements of the individuals in the study are shown in Table 1. The serum PTX3, VEGF and IL-8 values of the patient and control groups are shown in Table 2. A significant difference was found between the serum PTX3, VEGF and IL-8 values of the patient and control groups, with a lower mean of the patient group (Table 3). The serum PTX-3, VEGF and IL-8 values of the individuals enrolled in this study were correlated between the groups (Table 4). 

When all participants in the study were analyzed, irrespective of groups, high correlation was determined between serum PTX3 and VEGF, serum PTX3 and IL-8, and serum VEGF and IL-8 measurements (p<0.01; r=0.833, p<0.01; r=0.818, and p<0.01; r=0.803, respectively). High correlation was also determined between serum PTX3 and VEGF, serum PTX3 and IL-8, and serum VEGF and IL-8 measurements in the patient group (p<0.01; r=0.864, p<0.01; r=0.926, and p<0.01; r=0.854, respectively). In the control group, too high correlation was determined between serum PTX3 and VEGF, serum PTX3 and IL-8, and serum VEGF and IL-8 measurements (p<0.01; r=0.775, p<0.01; r=0.640, and p<0.01; r=0.561, respectively). Mean serum PTX3, VEGF andIL-8 values were lower in the patient group, but the difference was not statistically significant (p<0.05). 

**Table 1:** Serum PTX3, VEGF, and IL-8 Values of the Study Subjects

	No.	Minimum	Maximum	X±SD
PTX3 (ng/dl)	75	1.8	25.81	8,41±58.41±5,70
VEGF (ng/L)	75	89	14710	3271.44±3053.78
IIIL-8 (ng/L)	75	12.26	268.73	91.12±58.78

Values shown are mean ±standard deviation (SD). No.: Number of patients

**Table 2:** Serum PTX3, VEGF, and IL-8 Values for Groups

Group		No.	Minimum	Maximum	X±SDX
Patient	Serum PTX3 (ng/dl)	45	1.8	20.73	6.12±3.60
	Serum VEGF ( ng/L)	45	100.5	9825.5	1901.32±1689.23
	Serum IL-8 (ng/L)	45	12.26	212.86	64.18±40.47
Control	Serum PTX3 (ng/dl)	30	3.2	25.81	11.85±6.57
	Serum VEGF (ng/L)	30	89	14710	5326.62±3490.64
	Serum IL-8 (ng/L)	30	23.13	268.73	131.52±59.31

Values shown are mean ±standard deviation (SD). No, Number of patients

**Table 3:** A comparison of the Study Groups’ Serum PTX3, VEGF and IL-8 Values

	Patient	Control	p
	Mean± SD	Mean± SD	
Serum PTX3 (ng/dl)	6.12±3.60	11.85±6.57	0.001
VEGF (ng/L)	1901.32±1689.23	5326.61±3490.64	0.002
IL-8 (ng/L)	64.19±40.47	131.52±59.31	0.003

Values shown are mean ±standard deviation (SD). 1, 2, 3: Degree of significance between the patient and control groups. P <0.05 is statistical significant. Comparisons are made between control subjects and patients.

**Table 4:** Correlation between serum PTX3, VEGF, and IL-8 Values in the Patient and Control Groups

		Patient			Control		
		PTX3	VEGF	IL-8	PTX3	VEGF	IL-8
PTX3	r	1	0.864	0.926	1	0.775	0.64
	p	.	0	0	.	0	0
	n	30	30	30	45	45	45
VEGF	r	0.864	1	0.854	0.775	1	0.561
	p	0	.	0	0	.	0
	n	30	30	30	45	45	45
IL-8	r	0.926	0.854	1	0.64	0.561	1
	p	0	0	.	0	0	.
	n	30	30	30	45	45	45

P <0.05 is statistical significant. Comparisons are made between control subjects and patients

## Discussion

Since it generally manifests late, and has no specific symptoms, gastric cancer is usually detected at advanced stages. A significant proportion of cancer cases in the years ahead is expected to occur in less developed countries. Gastric cancer is twice as prevalent in men as in women. Men constituted 34 (75.55%) of the 45-member patient group in the present study, while 11 (24.45%) were women. The youngest patient in this study was 30, and the oldest was 84. Gastric cancer was most common in the sixth decade (60-69), in 20 patients (44.4%), with five patients (11.1%) being in their seventh decade, and three (6.6%) in their eighth decade. Five-year survival rates for gastric cancer when identified in the early stage is as high as 90%. 

Various factors are involved in the etiology of gastric cancer. Individuals with blood group A are known to be at greater risk. In one study, 331 (48.11%) of 688 gastric cancer patients were from blood group A (Edgren et al., 2010). Consistent with the previous literature, 27 (60%) of the 45 patients with gastric adenocarcinoma were from the A Rh (+) blood group, supporting the idea of individuals from this group being at greater risk. The fact that 42 (93.3%) of the patients in this study were members of the Rh (+) subgroup suggests a powerful association between gastric cancer and blood group. We think that further studies on this subject will contribute to our understanding of the pathogenesis of gastric cancer.

Several studies have indicated that PTX3 behaves as an oncosuppressor and may exhibit potential therapeutic effects in the treatment of cancer. PTX3 is capable of binding to different members of the fibroblast growth factor (FGF) family, thus inhibiting their biological effects. A study from 2009 found that overexpression of PTX3 reduced angiogenic activity in human breast carcinoma cells due to its capacity to neutralize FGF-2 (Margheri et al., 2009). PTX3 has been seen to prevent epithelial-mesenchymal changes induced by FGF-2 in human and mouse melanoma cells andalso to prevent metastatic potential by inhibiting FGF-2-mediated cell proliferation and angiogenesis (Ronca et al., 2013). In addition to PTX3 protein has been reported to act as an anti-angiogenic factor in multiplemyeloma and to exhibit cytotoxic effects against multiple myeloma cells by impairing signaling between plasma cells bone marrow-derived plasma cells and endothelial cells (Basile et al., 2013).

One previous study showed the antiangiogenic and antineoplastic activity of PTX3 in FGF/FGFR-dependent steroid hormone-related tumors (Leali et al., 2011; Ronca et al., 2013). A dramatic in vivo decrease has been reported in tumorigenic potential and angiogenic activity in tumor cells that overexpress PTX3. In line with its antitumoral activity, PTX3 immune reactivity that is normally abundant in the prostate gland has been shown to disappear in tumor biopsies taken from high-grade intraepithelial neoplasias and prostatic adenocarcinomasin experimental models of prostate cancer (Ronca et al., 2013). PTX3 exhibits antitumoralactivity when released from the tumor stroma. In another study, high *PTX3* overexpression was found in the circulation in transgenic mice driven by the endothelial Tiepromoter gene. Overproduction of PTX3 in these animals was found to powerfully inhibit tumoral growth, neovascularization, and metastatic activity of heterotropic, orthotropic, and autocrine models of FGF-dependent lung and prostate cancers. In contrast, the FGF-dependent metastatic and tumoral potentials of mouse melanoma cells of syngeneic PTX3-inoculated mice increased significantly (Ronca et al., 2015).

In the light of the above observations, PTX3 has been described as an extrinsic oncosuppressor that regulates complement-dependent inflammation (Bonavita et al., 2015). PTX3 deficiency has been reported to potentially raise the risk of cancer development by deregulating tumoral inflammation in various models of skin carcinogenesis. Absence of PTX3 release in mice inoculated with PTX3 has been shown to cause expansion of complement activation, increased macrophage infiltration, M2 differentiation, cytokine production, and angiogenesis. In parallel with these observation methylation-dependent epigenetic suppression in the *PTX* gene has been observed in some epithelial and mesenchymal human cancer series (Bonavita et al., 2015). This also corroborates the oncosuppressor role of PTX3. In particular, this information shows that the potential role of PTX3 in modulating the innate immune response in cancer is supported by experimental evidence. 

In a study involving six patients with esophageal squamous cell carcinoma, Wang et al., (2011) identified PTX3 as an epigenetically silenced gene in esophageal squamous cell carcinomas. *PTX3* expression has been shown to be downregulated in a large proportion of esophageal squamous cell carcinomas. Hypermethylation of the gene promoter is involved in such cancers. For that reason, and as also observed previously in prostate biopsies, immunohistochemical analysis of specimens taken from tumoral tissues has revealed a decrease in PTX3 immunoreactivity compared to normal tissues (Wang et al., 2011).

PTX3 binds to FGF2 with high affinity, thus inhibiting growth factor activity on target cells by preventing interaction between cell surface receptors and growth factor (Rusnati et al., 2004). In the light of all these findings the oncosuppressor potential of PTX3 derives from its effect on FGF and the complement-dependent pathway. In the present study, serum PTX3 levels were statistically significantly lower in the patient group. This further supports the idea, previously proposed in the literature, of an oncosuppressor effect of PTX3. We think that a deficit in PTX 3, which inhibits target cell activity by binding with high affinity to FGF2 in the gastric epithelium, thus preventing growth factor binding to receptors on the cell surface, prepares the foundation for gastric cancer development. 

The cytokine IL-8, a proinflammatory peptide, is principally released from monocytes, macrophages, keratinocytes, fibroblasts, and endothelial cells (Qazi et al., 2011). It affects functions more than growth in target cells and produced neutrophil activations and degranulation. Savage et al., (2006) reported no consistent association with the risk of gastric cancer in the Polish population. An epidemiological meta-analysisreported no direct association between *IL-8-251* gene polymorphisms and the risk of gastric cancer but that this can vary depending on the type of tumor, its location, *Helicobacter pylori* infection, ethnic origin, and from country to country (Liu et al., 2010). In the present study, mean serum IL-8 values were significantly higher in the control group compared to the patient group. We therefore anticipate thatserum IL-8 levels that vary depending on ethnicity and country, will be lower in gastric cancers in Turkey. We think that there is an association between defect or decrease in in PTX3, which acts as an oncosuppressor with anti-angiogenic and antineoplastic activities, and serum IL-8. When all participants were considered together, irrespective of groups, in the present study, high correlation was detected between serum PTX3 and IL-8 measurements. High correlation was also determined between serum PTX3 and IL-8 values in the patient group. In the light of these correlations, there exists a strong probability that low IL-8 is related to the effect of PTX3 on biochemical and molecular pathways.

The VEGF family, a member of the platelet-derived growth factor super-family, has important effects specific to endothelial cells. Members of the family are involved in several physiological and also pathological events. VEGF selectively plays important roles in morphogenesis and chemotaxis, in addition to stimulating vascular development with a mitogenic effect in endothelial cells (Bikfalvi, 2004).

One previous study determined higher serum VEGF-C levels in patients with lymph node metastasis and distant metastasis (WANG et al., 2012). Similarly, Tsirlis et al. observed lower VEGF-C levels in the preoperative period in a patient group, while VEGF-D levels were higher, and that VEGF-C levels increased while VEGF-D levels decreased in the postoperative period. Those authors describedserum VEGF-C and VEGF-D levels as statistically significant determinants of the presence of gastric cancer and showed that the VEGF-C/VEGF-D ration was a powerful determinant of malignancy (Tsirlis et al., 2010).

Vidal et al., (2009) observed significantly higher preoperative serum VEGF levels in patients with gastric cancer compared to a control group. Higher preoperative serum VEGF levels were shown to be associated with advanced disease (advanced TNM stage, perineural invasion, and a high lymph node ratio), a lower probability of recurrence-free status, and shorter disease-specific survival. Bilgiç and Tez (2015) reported that serum VEGF levels were associated with the type of tumor, its classification, and presence of distant tissue invasion.

In a study involving COPD patients, Yasuo et al., (2011) detected a decrease in VEGF levels in COPD patients and associated this withhypoxiainducible factor-1α (HIF-1α) transcript factor. In the present study, mean serum VEGF values were lower in the patient group compared to the control group. We also think that this suppression of VEGF is due to the effect of transcript factors such as HIF-1α and proteolytic enzymes. 

Shi and Wei (2016) suggested thatIL-8 may be a powerful promoter in gastric cancer angiogenesis by stimulating VEGF-A, VEGFR-1 and VEGFR-2 protein and mRNA release. Konno et al., (2003) observed higherplasma VEGF and IL-8 levels in a patient group. High IL-8 levels, tumors larger than 4 cm, tumors exhibiting deep invasion, lymph node involvement, and vascular invasion have been linked to shorter disease-free survival. In the present study, both serum VEGF and serum IL-8 levels were lower in the patient group and exhibited high correlation.

The current data show that levels of PTX3, IL-8, and VRGF that are normally independent, affect one another in gastric adenocarcinomasHowever, further molecular and genetic studies are now needed to explain the potential regulation mechanism between them. 

As a conclusion, serum PTX3, IL-8 and VEGF were significantly lower in the patient group in this study than in the control group. We think that a deficit in or suppression of PTX3, which behaves as an oncosuppressor, can play a role in the development of gastric adenocarcinoma. Levels of IL-8, a proinflammatory cytokine, can vary depending on the tumor type and location, whether accompanying *H. pylori* infection is present ethnicity, and country, and are low in gastric cancers in Turkey. Levels of VEGF, involved in several forms of physiological and pathological angiogenesis, were significantly lower in the patient group in the present study.

Correlation tests in the present study, irrespective of groups, show that serum PTX3, IL-8 and VEGF affect one another in gastric adenocarcinomas. However, further molecular and genetic studies are now needed to explain the potential regulation mechanism between them.

## Author Contribution Statement

Concept:M.Y; Design: E.K., M.Y.; Supervision: E.K,; Resource: M.Y., E.K. N.A, S.K., M.K..; Materials: M.Y., M.K.; Data: M.Y.; Analysis: S. A,.; Literature search: M.Y., E.K. N.A, S.K., M.K.; Writing: M.Y., E.K. N.A, S.K., M.K..; Critical revision: M.Y., E.K. N.A, S.K., M.K.
